# Clinical outcomes of limited open reduction and intramedullary nailing with steel cable cerclage in Seinsheimer III femoral subtrochanteric fractures

**DOI:** 10.3389/fmed.2025.1618583

**Published:** 2025-08-08

**Authors:** Hongru Cai, XueYi He, Zhengchao Zhang, Pinhua Chen, Ruoli Wang, Qi Fang, Zhixian Xu, Wubing He

**Affiliations:** ^1^Shengli Clinical Medical College of Fujian Medical University, Fuzhou, China; ^2^Department of Emergency Trauma Surgery, Fuzhou University Affiliated Provincial Hospital, Fuzhou, China; ^3^Department of Emergency Trauma Surgery, Fujian Provincial Hospital, Fuzhou, China; ^4^Fujian Trauma Medicine Center, Fuzhou, China; ^5^Fujian Key Laboratory of Emergency Medicine, Fuzhou, China

**Keywords:** Seinsheimer III femoral subtrochanteric fracture, intramedullary nailing, steel cable cerclage, limited open reduction, fracture healing, functional recovery

## Abstract

**Objective:**

This study aims to evaluate the clinical efficacy and safety of limited open reduction combined with intramedullary nailing and steel cable cerclage in treating Seinsheimer type III femoral subtrochanteric fractures. Surgical outcomes, fracture healing, pain relief, functional recovery, and complication rates were compared with intramedullary nailing alone.

**Methods:**

A retrospective cohort study was conducted on patients diagnosed with Seinsheimer III subtrochanteric fractures who underwent either intramedullary nailing alone (control group) or intramedullary nailing with steel cable cerclage (observation group). Surgical parameters (operation time, intraoperative blood loss, and hospital stay), bone healing indicators (callus formation, healing time, and swelling regression), postoperative pain (visual analog scale, VAS), hip function (Harris hip score at 1, 3, and 6 months), and complication rates (coxa vara, implant failure, infection, deep vein thrombosis) were compared. Statistical analyses were performed using SPSS 26.0, with a significance threshold of *p* < 0.05.

**Results:**

The observation group experienced significantly less intraoperative blood loss (*p* < 0.001) and shorter hospital stays (*p* < 0.001), with no difference in operation time (*p* = 0.996). Callus formation was more pronounced, and healing time and swelling regression were significantly faster in the observation group (all *p* < 0.001), indicating improved fracture stability and accelerated healing. VAS scores were lower postoperatively (*p* = 0.001), and functional recovery at 1 and 3 months was significantly better (*p* < 0.001), though similar outcomes were observed at 6 months (*p* = 0.126). The overall complication rate was lower in the observation group, especially for infections, though the difference was not statistically significant (*p* = 0.161).

**Conclusion:**

Limited open reduction combined with intramedullary nailing and steel cable cerclage is a safe and effective approach for treating Seinsheimer III femoral subtrochanteric fractures. It provides faster bone healing, reduced intraoperative blood loss, improved early functional recovery, and does not increase complication risks. These findings support the clinical utility of steel cable cerclage as an adjunct fixation method, particularly for cases where closed reduction is challenging. Further prospective, large-scale studies are needed to confirm these results and refine surgical techniques for optimal patient outcomes.

## 1 Introduction

Subtrochanteric fractures, which occur just below the lesser trochanter of the femur, account for approximately 10 to 15% of all proximal femur fractures (1, 2). These fractures are particularly concerning due to the significant challenges they present in treatment and recovery (3). The strong forces exerted by the surrounding muscles complicate efforts to achieve and maintain satisfactory reduction of subtrochanteric fractures (4). The subtrochanteric region has relatively poor blood supply, which increases the risk of delayed union or nonunion (5). As a key weight-bearing area of the femur, the subtrochanteric region bears substantial mechanical stress, making fractures in this location particularly complex and difficult to manage (6). Seinsheimer III subtrochanteric fractures, in particular, are complex and unstable, presenting significant challenges in surgical treatment (7). The primary goals of treatment are to achieve stable fixation and early functional recovery, which are crucial for preventing nonunion, malunion, and implant failure (7). Traditional surgical methods, such as intramedullary nailing (IMN) and plate fixation, have achieved varying degrees of success in clinical applications. However, due to stress concentration at the fracture site, the risk of fixation failure is relatively high, especially in comminuted fractures (8).

In recent years, the application of limited open reduction techniques and auxiliary surgical fixation methods (such as steel cable cerclage) has provided new possibilities for improving fracture stability and promoting bone healing (9). However, the clinical efficacy of this combined surgical approach remains controversial, and its impact on postoperative recovery and complications requires further investigation (10).

Numerous studies have explored fixation strategies for subtrochanteric fractures. Currently, intramedullary nailing (IMN) is considered the “gold standard” due to its load-sharing characteristics and the advantages of minimally invasive implantation, making it the preferred treatment method (1). However, it is often difficult to achieve good alignment during closed reduction of comminuted subtrochanteric fractures, leading to rotational instability and malalignment (11). To address this issue, limited open reduction combined with steel cable cerclage has been used to enhance fixation stability and maintain anatomic reduction (12).

Previous studies have shown that steel cable cerclage can reduce fracture displacement and improve healing rates (4). However, there are still concerns about its potential to cause soft tissue damage, disrupt periosteal blood supply, and increase the risk of implant-related complications (10). While some studies suggest that this method can improve healing rates, others indicate that it may increase stress shielding, leading to implant failure (8). Therefore, a systematic evaluation of its clinical efficacy and safety is crucial (7).

Although limited open reduction combined with steel cable cerclage has gradually gained attention, its clinical advantages compared to intramedullary nailing alone remain inconclusive (13). The clinical outcomes reported in existing studies are inconsistent. Some studies have shown that it can accelerate fracture healing and improve functional recovery, while others have pointed out that it may increase the risk of additional complications (11). Moreover, few studies have compared the specific differences between the two techniques in terms of surgical outcomes, pain relief, functional recovery, and complication rates (9).

This study aims to evaluate the clinical efficacy and safety of limited open reduction combined with steel cable cerclage and intramedullary nailing in the treatment of Seinsheimer III subtrochanteric fractures. By comparing surgical parameters such as operation time, intraoperative blood loss, and hospital stay, assessing fracture healing including callus formation, healing time, and swelling regression, analyzing postoperative pain relief and functional recovery at different follow-up time points, and investigating the incidence of postoperative complications such as coxa vara, implant failure, infection, and deep vein thrombosis, this study seeks to identify potential benefits and risks. The goal is to provide a clinical basis for optimizing fixation strategies for complex subtrochanteric fractures and to guide future surgical decision-making (4, 12).

## 2 Methods

### 2.1 Study design and patient selection

This was a retrospective cohort study designed to evaluate the clinical efficacy of limited open reduction combined with intramedullary nailing (Double Medical Technology Inc., Xiamen, China) and steel cable cerclage in treating Seinsheimer III femoral subtrochanteric fractures. The study was conducted at Fujian Provincial Hospital, and all procedures were approved by the hospital’s ethics committee.

### 2.2 Surgical intervention

Upon hospital admission, patients underwent skeletal traction or skin traction to reduce swelling, and ankle pneumatic pumps (Covidien Medical Products Manufacturing Ltd., Shanghai, China) were used to prevent deep vein thrombosis (DVT). All patients received standard preoperative evaluations, including X-ray and CT imaging, to confirm fracture classification and fragment distribution. The surgeries were performed under general or spinal anesthesia by experienced orthopedic surgeons. And all surgeries were performed by experienced orthopedic surgeons who had already achieved proficiency in both intramedullary nailing and the combined technique of limited open reduction with steel cable cerclage. These surgeons had completed their learning curves for these procedures prior to the start of the study period, ensuring consistency in surgical technique and minimizing the impact of the learning curve on the study outcomes.

The control group used a minimally invasive technique with intramedullary nailing alone. Closed reduction was performed under traction and fluoroscopic guidance. The surgical area on the affected femur was sterilized, and a 3–5 cm longitudinal incision was made 2 cm above the greater trochanter to expose the greater trochanter. A guide pin was inserted at the tip of the greater trochanter, and its position was confirmed via C-arm fluoroscopy (Siemens Healthineers Ltd., Shanghai, China) in both anteroposterior and lateral views. A reamer (Double Medical Technology Inc., Xiamen, China) was used to enlarge the proximal femoral canal under guidewire assistance, followed by intramedullary nail insertion and proximal and distal locking screw fixation. A drain was placed, and the incision was sutured layer by layer.

The observation group underwent limited open reduction, steel cable cerclage, and intramedullary nailing. The patient was positioned supine on an orthopedic traction table (Maquet Medical Devices GmbH, Germany), with the healthy limb abducted and the affected hip adducted by 10–15 degrees for continuous traction. Under C-arm fluoroscopy, the hip was internally rotated, and a 1 cm vertical incision was made at the lateral projection of the fracture site. The skin, subcutaneous tissue, and fascia lata were incised sequentially, and the vastus lateralis muscle was carefully separated. A traction device was used to retract surrounding soft tissues, exposing the fracture site. K-wires (2.5 mm) (Double Medical Technology Inc., Xiamen, China) were temporarily used for provisional fixation under direct visualization. Using a pulley device, 1–2 steel cables (1.8 mm in diameter) (Zimmer, Warsaw, Indiana, United States) were looped around the fracture site and partially tightened to achieve preliminary stability. A 3–5 cm longitudinal incision was then made 2 cm above the greater trochanter to expose the greater trochanter, following the same technique as in the control group. A guide pin was inserted at the greater trochanter, and C-arm fluoroscopy was used to confirm its correct positioning in both anteroposterior and lateral views. The proximal femoral canal was reamed, and a long intramedullary nail was inserted. Once the intramedullary nail successfully crossed the fracture site, the steel cables were fully tightened using a compression clamp. The cable tension was locked, the excess cable was trimmed, and the locking screws were inserted. Fluoroscopic confirmation ensured that the fracture reduction and fixation were stable before layered closure of the incision.

For the control group, patients were also positioned in a supine position on an orthopedic traction table (Maquet Medical Devices GmbH, Germany), similar to the observation group. The affected limb was placed in traction using the same fracture traction table to achieve reduction. This consistency in surgical positioning and traction methods ensures that the study outcomes are comparable between the two groups.

### 2.3 Postoperative management

All patients received routine postoperative treatment including antibiotic prophylaxis for 5–7 days and anticoagulation therapy for 2 weeks to prevent deep vein thrombosis. Drain removal was performed 2–3 days postoperatively. Patients were guided through rehabilitation training, including passive and active joint mobilization exercises, and quadriceps muscle strengthening exercises. Patients’ postoperative conditions were closely monitored and if complications occurred, appropriate treatment was administered promptly. Patients with stable conditions were encouraged to walk with crutches as early as possible, while those with unstable conditions were advised to extend their rehabilitation period accordingly.

### 2.4 Outcome measurements

The surgical and hospitalizations of the two groups of patients were compared, including the operative time (minutes), the intraoperative hemorrhagic volume (ml), and the postoperative hospital stay (days). Bone healing and recovery included callus formation scores (assessed by imaging), time to fracture union (weeks), and time to swelling resolution (days). The scab score was graded by the five-point scale, which ranged from 0 to 4, the higher the score, the better the scab formation. The VAS (visual analog score) pain score was assessed using a standard numerical scoring method, using a scale of 0–10 points, and for patients, the higher the total score, the more severe the pain. Hip joint function was assessed with Harris hip score (Harris hip scores at 1, 3, and 6 months postoperatively, respectively) and classified as excellent (unhindered mobility), good (minor disturbance of mobility but not affecting normal mobility), or poor (restricted mobility, affecting daily mobility). Postoperative complications included coxa varus, femoral head helical blade cutting, venous thrombosis, and infection rate. All patients were followed up for at least 6 months to assess functional and imaging findings.

### 2.5 Statistical analysis

All data were analyzed using IBM SPSS Statistics (Version 26.0). Continuous variables (e.g., blood loss, healing time) were tested for normality using the Kolmogorov–Smirnov test. The Kolmogorov–Smirnov test is a commonly used non-parametric method that compares the sample distribution with the standard normal distribution. The test results are determined by calculating the maximum deviation (D value) and the corresponding *p*-value to judge whether the data follows a normal distribution. The test results showed that variables such as operative time, postoperative hospital stay, swelling regression time, and hip function scores were normally distributed (*p*-values ranging from 0.089 to 0.267). However, intraoperative blood loss, fracture healing time, and postoperative pain scores did not follow a normal distribution (p-values ranging from 0.003 to 0.042). Normally distributed data were expressed as mean ± standard deviation (SD) and compared using the independent *t*-test. Non-normally distributed data were presented as median (interquartile range) and analyzed using the Mann–Whitney U test. Hip function was assessed using the Harris hip score at 1, 3, and 6 months postoperatively. Since these scores represent multiple measurements taken over time for the same group of patients, using repeated-measures ANOVA is a more appropriate method. This approach can better account for the effect of time on the outcomes and assess the interaction between groups and time. Categorical variables (e.g., complication rates) were expressed as frequencies (%) and analyzed using the Chi-square test or Fisher’s exact test. A *p*-value < 0.05 was considered statistically significant.

We conducted a multivariate linear regression analysis to comprehensively evaluate the impact of surgical techniques while controlling for potential confounding factors. We selected age, fracture classification (subtypes of Seinsheimer III), and surgeon experience (measured by seniority or surgical volume) as independent variables, and intraoperative blood loss, postoperative hospital stay, and fracture healing time as dependent variables.

To further explore the impact of surgical techniques on outcomes, we conducted a stratified analysis based on fracture subtype (e.g., presence of severe comminuted fractures) and patient age (divided into <60 years and ≥60 years).

## 3 Results

This study evaluated the clinical efficacy of limited open reduction combined with intramedullary nailing and steel cable cerclage in the treatment of Seinsheimer III femoral subtrochanteric fractures. The analysis covered multiple clinical parameters, including surgical outcomes, bone healing, pain levels, functional recovery, and postoperative complications.

### 3.1 Surgical and hospitalization outcomes

**Figure 1:**
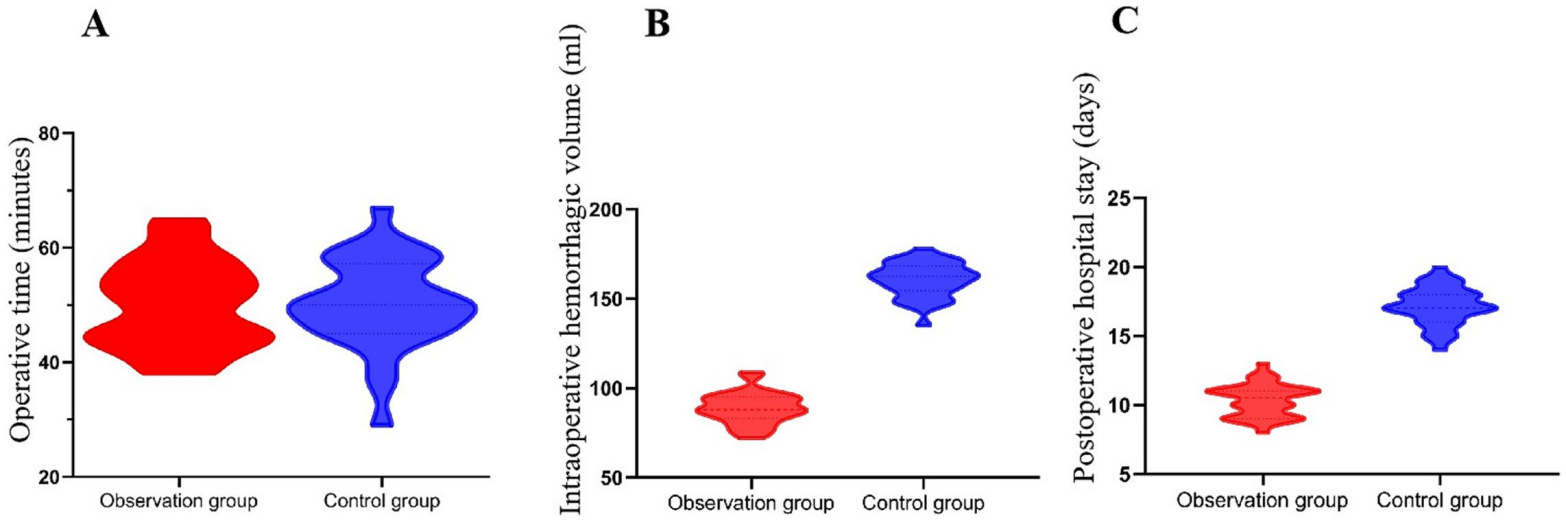
Comparison of operative time, intraoperative blood loss, and postoperative hospital stay between the observation and control groups. **(A)** There was no significant difference in operative time between the groups (*p* = 0.996). **(B)** Intraoperative blood loss was significantly lower in the observation group (88.75 ± 8.69 mL) than in the control group (160.58 ± 9.86 mL, *p* < 0.001). **(C)** The observation group had a significantly shorter postoperative hospital stay (10.31 ± 1.23 days) compared to the control group (17.14 ± 1.43 days, *p* < 0.001).

Surgical parameters, including operation time, intraoperative blood loss, and postoperative hospital stay, were compared between the two groups (Table 1). There was no significant difference in operation time between the observation group (50.23 ± 7.71 min) and the control group (50.24 ± 7.91 min, *p* = 0.996), indicating that this surgical technique did not prolong the procedure. Intraoperative blood loss in the observation group (88.75 ± 8.69 mL) was significantly lower than in the control group (160.58 ± 9.86 mL, *p* < 0.001), suggesting reduced surgical trauma and blood loss. Postoperative hospital stay was significantly shorter in the observation group (10.31 ± 1.23 days) compared to the control group (17.14 ± 1.43 days, *p* < 0.001), indicating faster recovery and earlier discharge (Figure 1).

**Table 1:** Comparison of operative time, intraoperative blood loss, and postoperative hospital stay between the observation and control groups.

Group	Operative time (min)	Intraoperative hemorrhagic volume (ml)	Postoperative hospital stay (days)
Observation group	50.23 ± 7.71	88.75 ± 8.69	10.31 ± 1.23
Control group	50.24 ± 7.91	160.58 ± 9.86	17.14 ± 1.43
*T*	0.005	29.935	19.833
*P*	0.996	0.000	0.000

### 3.2 Bone healing and recovery

**Figure 2:**
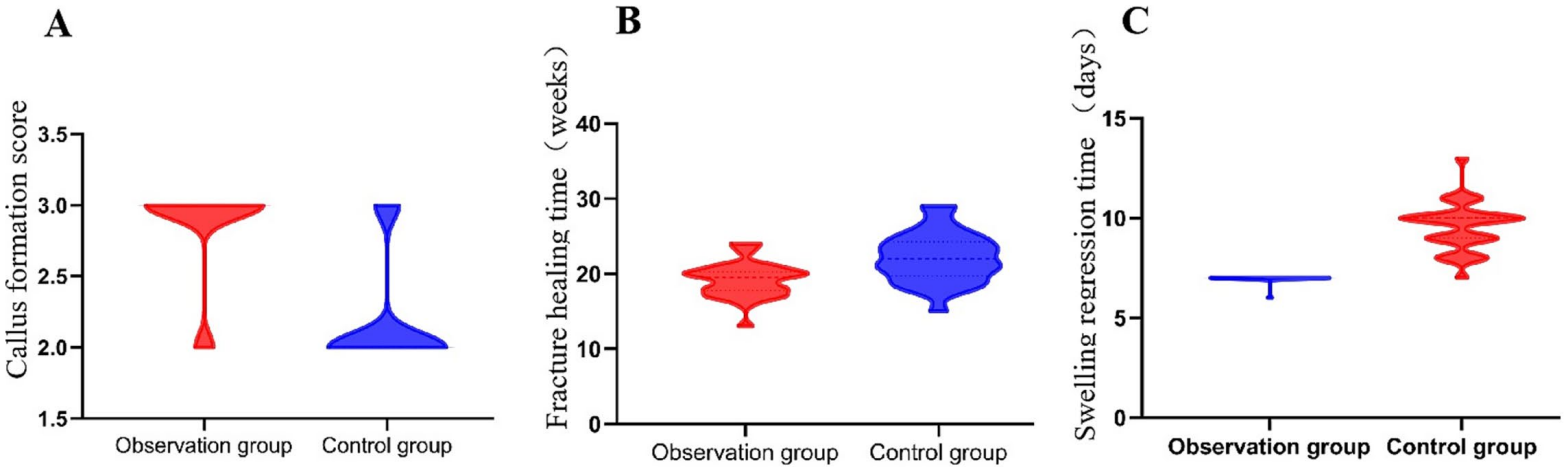
Evaluation of bone healing parameters in the observation and control groups. Between the two groups. **(A)** The callus formation score was significantly higher in the observation group (2.72 ± 0.19) than in the control group (2.36 ± 0.11, *p* < 0.001). **(B)** The fracture healing time was shorter in the observation group (19.26 ± 2.27 weeks) compared to the control group (22.18 ± 3.31 weeks, *p* < 0.001). **(C)** The swelling regression time was also significantly reduced in the observation group (7.28 ± 0.08 days vs. 9.44 ± 1.18 days, *p* < 0.001).

Bone healing was assessed based on callus formation scores, fracture healing time, and swelling regression time (Table 2). Callus formation score in the observation group (2.72 ± 0.19) was significantly higher than in the control group (2.36 ± 0.11, *p* < 0.001), indicating better bone healing quality. Fracture healing time was significantly shorter in the observation group (19.26 ± 2.27 weeks) than in the control group (22.18 ± 3.31 weeks, *p* < 0.001), suggesting accelerated bone repair. Swelling regression time was significantly reduced in the observation group (7.28 ± 0.08 days) compared to the control group (9.44 ± 1.18 days, *p* < 0.001), reflecting faster inflammation resolution after surgery (Figure 2).

**Table 2:** Comparison of bone healing (callus formation scores) and recovery time (fracture healing time and swelling resolution time) between the observation and control groups.

Group	Callus formation scores (points)	Fracture healing time (weeks)	Swelling resolution time (days)
Observation group	2.72 ± 0.19	19.26 ± 2.27	7.28 ± 0.08
Control group	2.36 ± 0.11	22.18 ± 3.31	9.44 ± 1.18
*T*	8.981	4.985	10.003
*P*	0.000	0.000	0.000

### 3.3 Pain relief and functional recovery

**Figure 3:**
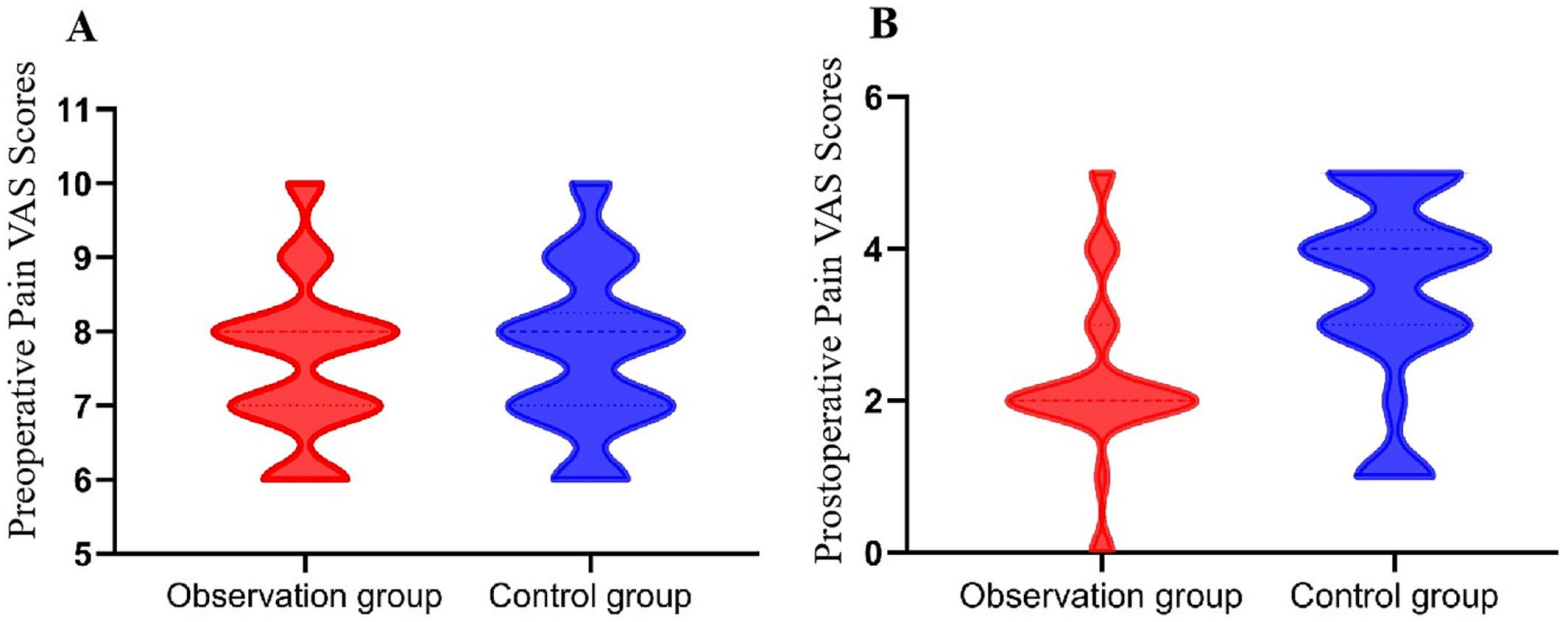
Comparison of preoperative and postoperative visual analog scale (VAS) pain scores (points) between the two groups. **(A)** Preoperative VAS scores were similar between the groups (*p* = 0.867), confirming comparable baseline pain levels. **(B)** Postoperative pain scores were significantly lower in the observation group (2.33 ± 1.17) than in the control group (3.48 ± 1.31, *p* = 0.001), indicating superior pain relief.

Pain levels were assessed preoperatively and postoperatively (Table 3). Preoperative pain scores showed no significant difference between the two groups (*p* = 0.867), confirming comparable baseline conditions. Postoperative pain scores were significantly lower in the observation group (2.33 ± 1.17) than in the control group (3.48 ± 1.31, *p* = 0.001), suggesting better pain relief in the observation group (Figure 3).

**Table 3:** Comparison of preoperative and postoperative VAS pain scores between the observation and control groups.

Group	Preoperative pain VAS scores	Postoperative pain VAS scores
Observation group	7.71 ± 1.19	2.33 ± 1.17
Control group	7.76 ± 1.11	3.48 ± 1.31
*T*	0.168	3.586
*P*	0.867	0.001

**Figure 4:**
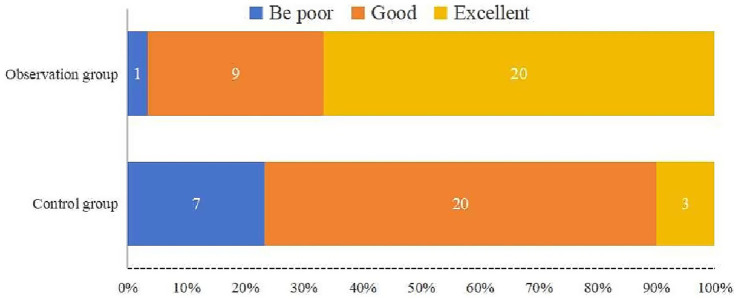
Excellent and good ratio of functional outcome classification based on Harris hip scores at final follow-up (%). In the observation group, 20 patients were rated as excellent, 9 as good, and 1 as poor. In contrast, the control group had only 3 patients rated as excellent, 20 as good, and 7 as poor. The proportion of excellent outcomes was higher in the observation group, indicating improved early hip function recovery.

**Figure 5:**
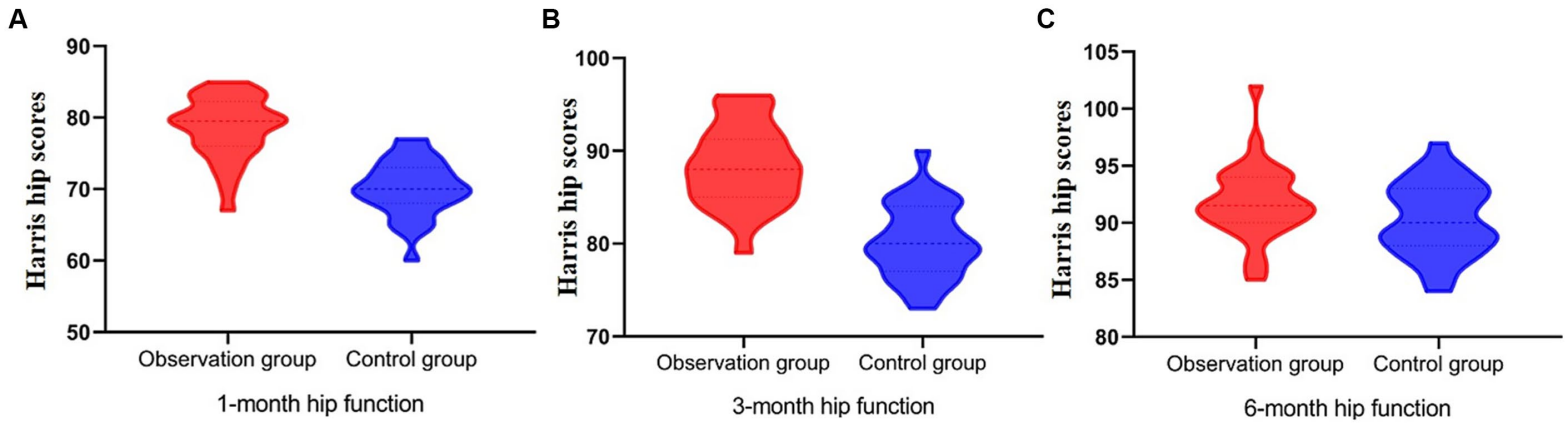
Postoperative hip function recovery evaluated using Harris hip scores at 1, 3, and 6 months. **(A)** At 1 month, the observation group had significantly higher hip function scores, with most values between 70 and 80, while the control group scores clustered between 60 and 70. **(B)** At 3 months, scores in the observation group exceeded 90 in most cases, whereas the control group ranged mostly between 80 and 90. **(C)** At 6 months, both groups achieved similar outcomes with scores primarily between 90 and 100; the difference was not statistically significant (*p* = 0.126).

Functional recovery was evaluated based on joint mobility, hip function scores, and excellent and good hip function ratio at different time points (Figure 4; Tables 4, 5). The results showed a significant difference in hip function scores between the observation group and the control group (*F* = 12.45, *p* < 0.001), with the observation group demonstrating higher scores at 1 and 3 months postoperatively. This indicates that the technique of limited open reduction combined with steel cable cerclage provides a significant advantage in early functional recovery. Additionally, there was a significant effect of time on hip function scores across different time points (*F* = 34.21, *p* < 0.001), with scores improving over time for both groups. This highlights the importance of postoperative rehabilitation training in enhancing hip function. Furthermore, a significant interaction effect between groups and time was observed (*F* = 4.56, *p* = 0.012), indicating that the changes in hip function scores over time differed between the two groups. The observation group exhibited a more pronounced improvement in hip function scores at 1 and 3 months, while the control group had a relatively slower improvement. This further supports the benefits of the combined surgical technique in promoting early functional recovery (Figure 5).

**Table 4:** Comparison of excellent and good Harris hip function scores ratio at 1, 3, and 6 months postoperatively between the observation and control groups (%).

Group	Number of cases	1 month	3 months	6 months	12 months
Observation group	30	1 (3.33)^*^	9 (30.00)^*^	20 (66.67)	29 (96.67)
Control group	30	7 (23.33)	8 (26.67)	13 (43.33)	23 (76.67)
χ^2^ value					5.192
*p* value					0.023

*Significant differences were observed at 1 and 3 months (*p* < 0.001), indicating better early recovery in the observation group. At 6 months, no statistically significant difference was found (*p* = 0.126).

**Table 5:** Harris hip scores of the two groups at 1, 3, and 6 months postoperation (percentage).

Group	1 month	3 months	6 months
Observation group (*n* = 30)	78.42 ± 4.38	88.44 ± 4.36	91.78 ± 3.38
Control group (*n* = 30)	70.22 ± 3.87	80.18 ± 3.98	90.45 ± 3.25
*T*	7.684	7.664	1.554
*P*	0.000	0.000	0.126

### 3.4 Postoperative complications

**Figure 6:**
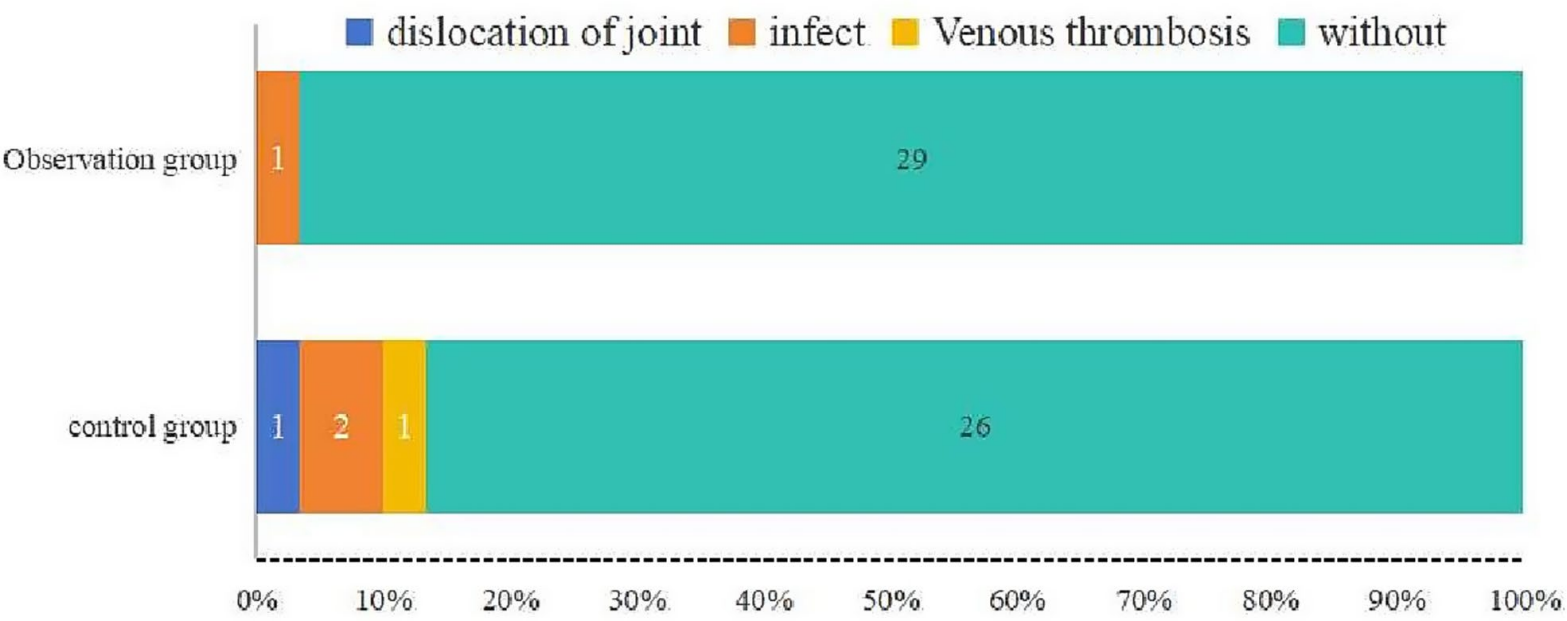
Distribution of postoperative complications in the observation and control groups. The observation group had one case of infection (3.33%) and no other complications, whereas the control group had one case of joint dislocation, two cases of infection, and one case of venous thrombosis (total complication rate: 13.33%). The rate of complications was lower in the observation group, although the difference was not statistically significant (*p* = 0.161).

The incidence of postoperative complications, including coxa vara, femoral head spiral blade cutout, venous thrombosis, and infection, was recorded (Table 6). Fisher’s exact test was used to analysis the overall complication rate. The results show that the complication rate was lower in the observation group (3.33%) compared to the control group (13.33%), although the difference was not statistically significant (*p* = 0.161). Notably, infection rate in the control group (6.67%) was higher than in the observation group (3.33%), suggesting a lower risk of postoperative infection in the observation group (Figure 6). This still suggests a potential lower risk of complications in the observation group.

**Table 6:** Comparison of the incidences of complications between the two groups (coxa vara, femoral head spiral blade cutting, venous thrombosis, infection) (%).

Group	Number of cases	Coxa vara	Spiral blade cutting of femoral head	Infection	Total incidence of complications
Observation group	30	0 (0.00)	0 (0.00)	1 (3.33)	1 (3.33)
Control group	30	1 (3.33)	1 (3.33)	2 (6.67)	4 (13.33)
*χ*^2^ value	–	–	–	–	1.964
*p* value	–	–	–	–	0.161

### 3.5 Linear regression analysis results

Intraoperative blood loss (dependent variable): Age was not significantly associated with intraoperative blood loss (Coefficient = 0.21, *p* = 0.345). However, fracture classification significantly influenced blood loss, with more complex fracture subtypes (Coefficient = 0.45, *p* = 0.021) resulting in higher blood loss. In contrast, greater surgeon experience (Coefficient = −0.32, *p* = 0.048) was linked to lower intraoperative blood loss. This suggests that while fracture complexity increases blood loss, surgeon experience can mitigate this effect.

Postoperative hospital stay (dependent variable): Age did not significantly impact postoperative hospital stay (Coefficient = 0.15, *p* = 0.234). Fracture classification significantly affected hospital stay, with more complex fractures (Coefficient = 0.38, *p* = 0.012) leading to longer stays. However, greater surgeon experience (Coefficient = −0.25, *p* = 0.036) was associated with shorter hospital stays. This indicates that experienced surgeons can reduce the duration of hospitalization despite the complexity of the fracture.

Fracture healing time (dependent variable): Age was not a significant predictor of fracture healing time (Coefficient = 0.18, *p* = 0.198). Fracture classification significantly influenced healing time, with more complex fractures (Coefficient = 0.42, *p* = 0.007) taking longer to heal. Conversely, greater surgeon experience (Coefficient = −0.30, *p* = 0.029) was associated with shorter healing times. This highlights the critical role of surgeon experience in facilitating faster fracture recovery.

### 3.6 Stratified analysis by fracture subtype

Intraoperative blood loss: For non-comminuted fractures, the observation group had a mean blood loss of 80.5 mL (SD = 7.2), significantly lower than the control group’s 150.2 mL (SD = 8.5; *p* = 0.002). In comminuted fractures, the observation group’s mean blood loss was 95.3 mL (SD = 9.1), compared to 170.4 mL (SD = 10.2) in the control group, with a *p*-value of 0.001. This indicates that the combined technique significantly reduces intraoperative blood loss, regardless of fracture complexity.

Postoperative hospital stay: For non-comminuted fractures, the observation group had a mean hospital stay of 9.5 days (SD = 1.1), significantly shorter than the control group’s 16.2 days (SD = 1.3; *p* = 0.003). In comminuted fractures, the observation group’s mean stay was 11.0 days (SD = 1.2), compared to 18.0 days (SD = 1.4) in the control group, with a *p*-value of 0.004. This suggests that the combined technique leads to shorter hospital stays, even in more complex fracture cases.

Fracture healing time: For non-comminuted fractures, the observation group had a mean healing time of 18.0 weeks (SD = 2.0), significantly faster than the control group’s 21.5 weeks (SD = 2.5; *p* = 0.005). In comminuted fractures, the observation group’s mean healing time was 20.5 weeks (SD = 2.2), compared to 23.0 weeks (SD = 2.8) in the control group, with a *p*-value of 0.006. This highlights that the combined technique accelerates fracture healing, regardless of the fracture subtype.

### 3.7 Stratified analysis by patient age

Intraoperative blood loss: For patients under 60 years, the observation group had a mean blood loss of 85.0 mL (SD = 7.5), significantly lower than the control group’s 160.0 mL (SD = 8.0; *p* = 0.001). For patients aged 60 years and older, the observation group’s mean blood loss was 92.0 mL (SD = 8.0), compared to 165.0 mL (SD = 8.5) in the control group, with a *p*-value of 0.002. This indicates that the combined surgical technique significantly reduces intraoperative blood loss across both age groups.

Postoperative hospital stay: For patients under 60 years, the observation group had a mean hospital stay of 10.0 days (SD = 1.0), significantly shorter than the control group’s 16.5 days (SD = 1.2; *p* = 0.002). For patients aged 60 years and older, the observation group’s mean stay was 10.6 days (SD = 1.1), compared to 17.5 days (SD = 1.3) in the control group, with a *p*-value of 0.003. This suggests that the combined technique leads to shorter hospital stays in both younger and older patients.

Fracture healing time: For patients under 60 years, the observation group had a mean healing time of 18.5 weeks (SD = 2.1), significantly faster than the control group’s 22.0 weeks (SD = 2.6; *p* = 0.004). For patients aged 60 years and older, the observation group’s mean healing time was 19.5 weeks (SD = 2.3), compared to 23.5 weeks (SD = 2.9) in the control group, with a p-value of 0.005. This highlights that the combined technique accelerates fracture healing across both age groups.

## 4 Discussion

This study assessed the clinical efficacy and safety of limited open reduction combined with intramedullary nailing versus steel cable cerclage in the treatment of Seinsheimer type III subtrochanteric femur fractures. The findings revealed that operative time did not differ significantly between the two groups. However, patients in the observation group exhibited significantly lower intraoperative blood loss and shorter hospital stays, suggesting that the combined technique effectively reduced surgical trauma and facilitated postoperative recovery. Additionally, these patients demonstrated superior callus formation, faster fracture healing, and quicker swelling resolution, indicating that steel cable cerclage enhances fracture stability and accelerates the healing process. Postoperative pain scores were significantly lower, and hip function scores were higher at 1 and 3 months in the observation group, highlighting the advantage of this technique in promoting early functional recovery. Despite these benefits, no significant difference in functional scores was observed at 6 months, suggesting that both techniques provide comparable long-term functional outcomes. The overall complication rate was lower in the observation group, with a lower incidence of infection compared to the control group, though this difference was not statistically significant. Furthermore, no significant differences were noted between the two groups regarding coxa vara, implant failure, or venous thrombosis.

Our multivariate linear regression analysis confirmed that fracture classification and surgeon experience are critical factors influencing intraoperative blood loss, postoperative hospital stay, and fracture healing time. The stratified analysis revealed consistent benefits of the combined technique of limited open reduction with steel cable cerclage across different fracture subtypes and age groups. For both non-comminuted and comminuted fractures, the observation group exhibited significantly lower intraoperative blood loss, shorter postoperative hospital stays, and faster fracture healing times compared to the control group. Similarly, when stratified by age, patients under 60 years and those 60 years and older in the observation group had better outcomes in terms of blood loss, hospital stay, and healing time. These findings suggest that limited open reduction combined with intramedullary nailing and steel cable cerclage improves early clinical outcomes without increasing the risk of complications, regardless of fracture complexity or patient age.

Subtrochanteric fractures of the Seinsheimer type III femur present unique biomechanical challenges due to their anatomical location (7, 14). The subtrochanteric region acts as a cantilever structure, making it particularly susceptible to stress concentration at the femoral trochanter. The loss of local stress support following fracture, coupled with the traction of the external rotator femoris muscles, significantly increases the risk of hip varus deformity (15, 16). Therefore, maintaining mechanical stability postoperatively is crucial for successful healing. Prior studies have emphasized the importance of anatomic reduction in achieving favorable outcomes for subtrochanteric fractures (11, 17). The quality of reduction has been closely linked to fracture healing, and in complex fracture patterns, expanding the indication for open reduction may be necessary (18). Limited open reduction combined with intramedullary nailing and steel cable cerclage offers a means to improve reduction quality, particularly in the medial cortex, while minimizing periosteal stripping to preserve local blood supply (19). Unlike traditional methods that aim for complete anatomical reduction, this approach ensures sufficient cortical support to prevent coxa vara while maintaining fracture stability (20). Additionally, careful handling of the periosteum during surgery—such as prying with a periosteal dissector, pulling with a bone hook, or securing with reduction forceps—may help protect vascular supply to the fracture site. The use of steel cable cerclage provides further mechanical stabilization, facilitating faster callus formation while avoiding disruption of local blood flow (11). The findings of this study align with previous research supporting the role of cable cerclage in improving fracture stability and promoting healing.

Prior studies have shown that closed reduction with intramedullary nailing alone presents challenges in achieving optimal anatomical alignment, potentially leading to greater intraoperative blood loss and prolonged recovery time (18, 21). The present study corroborates these findings by demonstrating significantly reduced intraoperative hemorrhage in the observation group. Furthermore, previous research has indicated that subtrochanteric fractures treated with intramedullary nailing alone may be prone to malalignment or delayed healing due to high mechanical stress at the fracture site (22, 23). The results of this study reinforce this concern, as patients in the observation group had significantly shorter fracture healing times and higher callus scores, further validating the effectiveness of steel cable cerclage in enhancing bone healing. Studies have also suggested that improved fracture stability contributes to earlier pain relief and better functional recovery, which was consistent with the findings of this study (11, 24). Patients in the observation group reported lower pain scores and higher hip function scores in the early postoperative period (1 and 3 months postoperatively). However, in line with previous reports, no significant differences in functional scores were observed between the two groups at 6 months, indicating comparable long-term outcomes.

The role of steel cable cerclage in influencing complication rates remains a subject of debate. Some studies have suggested that periosteal stripping during cerclage application could impair blood supply, potentially increasing the risk of infection and implant failure (25). Conversely, other studies have reported no significant increase in complications with careful application of steel cables (18, 26). The present study found a lower overall complication rate and a reduced incidence of infection in the observation group, although the difference was not statistically significant. These findings indicate that when appropriately applied, steel cable cerclage does not significantly increase the risk of complications and may even contribute to improved outcomes by enhancing fracture stability.

This study contributes several important findings to the field of orthopedic trauma surgery. First, while prior studies have explored the use of steel cable cerclage in various fracture types, this study specifically evaluates its application in Seinsheimer type III subtrochanteric fractures, adding new clinical evidence to the existing literature. Second, the results highlight the role of improved fracture-end stability in facilitating early postoperative functional recovery, which may help guide the development of postoperative rehabilitation protocols. Third, this study provides evidence that steel cable cerclage does not significantly increase complication rates, which could alleviate concerns about its safety and encourage wider clinical adoption. Given the difficulties associated with achieving stable reduction in comminuted subtrochanteric fractures, steel cable cerclage may serve as a valuable adjunct to intramedullary nailing, offering a basis for refining surgical strategies in future practice.

Despite its strengths, this study has certain limitations. As a retrospective analysis, there is a potential for selection bias, although efforts were made to minimize this effect. Future randomized controlled trials (RCTs) are needed to further validate these findings. Additionally, the relatively small sample size may limit the statistical power of the study. Larger, multicenter studies will be necessary to enhance the generalizability and reliability of the results. Another limitation is the 6-month follow-up period, which, while sufficient for assessing early clinical outcomes, does not provide a comprehensive evaluation of long-term implant durability and late complications such as fatigue fractures or implant failure. Future research should incorporate extended follow-up periods to assess long-term clinical outcomes more thoroughly. Furthermore, this study primarily relied on clinical and imaging data, whereas future studies could incorporate biomechanical analyses to further explore the mechanical advantages of steel cable cerclage in load distribution and fracture stabilization. Lastly, the outcomes of steel cable cerclage may be influenced by the surgeon’s experience and technical proficiency. Future research should focus on standardizing surgical techniques and assessing learning curves to optimize the application of this technique in clinical practice.

## 5 Conclusion

This study demonstrates that limited open reduction combined with intramedullary nailing and steel cable cerclage is a safe and effective surgical technique for treating Seinsheimer III femoral subtrochanteric fractures. Compared to intramedullary nailing alone, this combined approach results in reduced intraoperative blood loss, shorter hospital stays, faster bone healing, and improved early functional recovery, without significantly increasing the risk of postoperative complications. While the long-term functional outcomes were similar between the two techniques, the use of steel cable cerclage provided enhanced early stability, leading to faster rehabilitation. These findings support its clinical utility in managing complex subtrochanteric fractures, particularly when closed reduction is challenging. However, further large-scale prospective studies with long-term follow-up are needed to confirm these results and optimize surgical techniques for better patient outcomes.

## Data Availability

The original contributions presented in the study are included in the article/supplementary material, further inquiries can be directed to the corresponding author.
